# Identification and Validation of Serum Biomarkers to Improve Colorectal Cancer Diagnosis

**DOI:** 10.1002/cam4.70460

**Published:** 2024-12-04

**Authors:** Minha Lea Yoon, Hyelim Chun, HyunJu Lee, WooJeong Seo, Jung Young Lee, Jung Hwan Yoon

**Affiliations:** ^1^ Clinical Trial Center Gangnam St. Peter's Hospital Seoul Republic of Korea; ^2^ Department of Pathology College of Medicine, The Catholic University of Korea Seoul Republic of Korea

**Keywords:** biomarkers, colorectal cancer, diagnosis, ELISA

## Abstract

**Background:**

The pressing need for reliable biomarkers in colorectal cancer (CRC) diagnosis and prognosis is a major global health concern. Current diagnostic methods rely heavily on invasive procedures like colonoscopy, and existing biomarkers such as Carbohydrate Antigen 19‐9 (CA19‐9) and Carcinoembryonic Antigen (CEA) exhibit limitations in accuracy and specificity.

**Aims:**

This study aims to identify and validate novel biomarkers that can enhance the early detection and diagnostic precision of CRC while overcoming the shortcomings of conventional biomarkers.

**Materials and Methods:**

Leveraging advancements in genomic and proteomic technologies, gene expression datasets were obtained from the Gene Expression Omnibus (GEO) and The Cancer Genome Atlas (TCGA). We identified differentially expressed genes (DEGs) and conducted further analyses, including Gene Ontology (GO) enrichment and Protein‐Protein Interaction (PPI) network construction. Five promising biomarkers—INHBA, MMP7, PSAT1, SLC7A5, and TGFBI—were selected based on their robust performance in Receiver Operating Characteristic (ROC) curve analysis. Serum concentrations of these biomarkers were measured in 200 CRC patients and 100 healthy controls.

**Results:**

The study revealed significantly elevated expression levels of the selected biomarkers in CRC tissues compared to normal tissues. Additionally, serum concentrations of INHBA, MMP7, PSAT1, SLC7A5, and TGFBI were notably higher in CRC patients than in healthy individuals, with Area Under the Curve (AUC) values ranging from 0.8361 to 0.9869 indicating high diagnostic accuracy. Optimal cutoff values for diagnosis ranged from 38.9 pg/mL to 280.7 pg/mL, yielding sensitivity and specificity values between 74.5% and 92.9%.

**Discussion:**

The findings underscore the potential of INHBA, MMP7, PSAT1, SLC7A5, and TGFBI as effective non‐invasive biomarkers for CRC detection. Their elevated serum concentrations and robust discriminatory abilities highlight their promise in improving diagnostic accuracy and patient outcomes compared to traditional biomarkers.

**Conclusion:**

The identification and validation of these novel biomarkers represent a significant advancement in CRC diagnosis and management. Further studies are required to validate their clinical applicability in larger cohorts and to elucidate their functional roles in CRC pathogenesis, ultimately enhancing diagnostic strategies and personalized treatment approaches.

## Graphical abstract

Recent research highlights five potential biomarkers—INHBA, MMP7, PSAT1, SLC7A5, and TGFBI—with elevated levels in colorectal cancer tissues and serum of patients, showing promise for non‐invasive CRC detection. These biomarkers exhibit high diagnostic accuracy, suggesting their potential to improve current diagnostic methods and enhance personalized treatment approaches, though further validation and functional studies are required for larger cohorts.

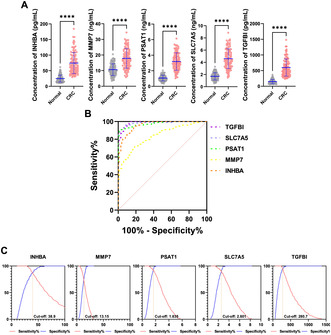

**Abbreviations**
AUC: area under the curve
CA19‐9: carbohydrate antigen 19‐9
CEA: carcinoembryonic antigen
CRC: colorectal cancer
DEGs: differentially expressed genes
DOR: diagnostic odds ratio
ELISA: enzyme‐linked immunosorbent assay
FNF: false‐negative fraction
FPF: false‐positive fraction
GEO: gene expression omnibus
GO: geneontology
LR: likelihood ratio
NPV: negative predictive value
PPI: protein–protein interaction
PPV: positive predictive value
ROC: receiver‐operator characteristic
TCGA: the cancer genome atlas
TNF: true negative fraction
TPF: true positive fraction

## 1 Introduction

Colorectal cancer (CRC) poses a substantial challenge to global health. Considered as one of the most prevalent worldwide malignancies, CRC is a leading contributor to cancer‐related morbidity and mortality [1]. The stage at which CRC is detected intimately shapes precise treatment modality and allows for more effective patient prognosis. Therefore, there is an urgent need to develop dependable biomarkers for CRC that are capable of facilitating early detection, precise diagnosis, and prognostic assessment.

Currently, the diagnostic approach for colon cancer primarily relies on an invasive procedure such as colonoscopy, which is costly, inconvenient, and carries a risk of complications despite its effectiveness [2]. Existing biomarkers for colon cancer, such as Carbohydrate Antigen 19‐9 (CA19‐9) and Carcinoembryonic Antigen (CEA), are also widely used as biomarkers for CRC diagnosis [3, 4, 5, 6]. However, both markers present definite limitations despite their utility in clinical practice. For example, CA19‐9, commonly used for pancreatic cancer, lacks the requisite specificity for accurate CRC diagnosis [7, 8]. CEA exhibits enhanced sensitivity but poses concerns regarding its specificity due to elevations observed in diverse non‐malignant conditions, ultimately limiting its diagnostic efficacy [9, 10, 11]. These deficiencies underscore the critical need for novel biomarkers capable of overcoming the shortcomings of existing markers to provide more reliable diagnostic and prognostic insights for CRC.

The present study's impetus emanates from the necessity to surmount the challenges posed by current CRC biomarkers. By utilizing the advancements in genomic and proteomic technologies, our investigation endeavors to identify and assess novel biomarkers exhibiting superior diagnostic performance compared to CA19‐9 and CEA. Ultimately, we aim to contribute towards developing a robust and precise diagnostic tool for CRC by scrutinizing potential biomarker candidates and delving into the molecular landscape of CRC.

## 2 Materials and Methods

### 2.1 Gene Expression Datasets

This research utilized Gene Expression Omnibus (GEO) (https://www.ncbi.nlm.nih.gov/geo/) and The Cancer Genome Atlas (TCGA), a free public functional genomics database including array‐ and sequence‐based data. Colorectal adenomas and CRC were main search terms. Datasets were screened according to the following criteria: (1) samples compared CRA/CRC and normal colorectal tissue, (2) human samples, (3) expression profile arrays, and (4) the number of samples in each group was greater than or equal to five reusable datasets for our analysis, and complied with relevant ethical regulations.

### 2.2 Identification and Integration of Common DEGs

The gene expression profiles were downloaded from the GEO and TCGA databases. Raw data from each dataset were processed using R statistical software (version 3.5.1). The analysis of screened Differentially Expressed Genes (DEGs) was carried out using the limma package. The RMA algorithm in the Affy package was used to preprocess data. The classical test was applied to identify DEGs. The adjusted *p* value < 0.05 and log_2_ fold change > 1 were considered cutoff values. Common DEGs from the datasets were integrated by Venn analysis.

### 2.3 GO Enrichment Analyses of Common DEGs

The characteristic biological attributes of common DEGs were identified using geneontology (GO) analysis (http://www.geneontology.org). The g:Profiler (https://biit.cs.ut.ee/gprofiler/), a free online tool for the functional classification of genes, was used to conduct GO (biological processes, cellular component, and molecular function). A *p* value < 0.05 was set as the cutoff criterion for these analyses.

### 2.4 Protein–Protein Interaction Network Construction and NetworkAnalyzer Analysis

A Protein–Protein Interaction (PPI) network of extracellular region and space DEGs was constructed using the Search Tool for the Retrieval of Interacting Gene (Genemania; https://genemania.org) database. Then, Cytoscape software was utilized to construct a protein interaction relationship network. NetworkAnalyzer software was used to calculate connectivity and identify hub genes.

### 2.5 Samples

Chonnam National University Hwasun Hospital, a member of the Korea Biobank Network, provided 60 anonymized frozen CRC tissues along with corresponding normal tissue samples. This study included human sera obtained from 200 patients diagnosed with CRC (average age: 62.7 ± 12.8; male: *n* = 129, female: *n* = 71) and 100 healthy controls (average age: 56.1 ± 4.76; male: *n* = 68, female: *n* = 32). To minimize institutional bias, healthy and patient samples were sourced from two National Biobanks: healthy controls from Seoul St. Mary's Hospital and 200 patients diagnosed with CRC from Chonnam National University Hwasun Hospital. All samples were collected with Institutional Review Board (IRB) approval. All participants provided written informed consent in accordance with the Declaration of Helsinki. Serum samples for both patients and healthy controls were collected from 2015 to 2020.

For healthy controls, serum samples were randomly collected from individuals without a history of cancer diagnosis, confirmed to be free of CRC and colitis through health examinations. Notably, there were no instances of familial cancer among the patients. The CRC stages among the patients were distributed as follows: 47 patients with stage I, 58 with stage II, 67 with stage III, and 28 with stage IV. Unfortunately, information on smoking status, alcohol consumption, and tumor location was not available from the two National Biobanks.

### 2.6 Real‐Time RT‐qPCR

RNA was extracted from the cells using the RNeasy kit by Qiagen, Valencia, CA, USA. The extracted RNA was then converted to cDNA using the Life Technologies kit in Carlsbad, CA, USA. For quantification, qPCR was conducted following standard protocols on a Bio‐Rad IQ5 real‐time PCR platform. As a control, the average mRNA expression value in non‐neoplastic colonic mucosa was used. The mRNA expression change in each case was subsequently normalized to the mean value in non‐neoplastic colonic mucosa. A decrease or increase in mRNA expression was indicated by changes less than 0.5‐fold or greater than 1.5‐fold, respectively. All primer details can be found in the Table S1.

### 2.7 Measurement of INHBA, MMP7, PSAT1, SLC7A5, and TGFBI Protein Concentrations in Colorectal Cancer Tissues

INHBA, MMP7, PSAT1, SLC7A5, and TGFBI protein expression levels in CRC tissues were analyzed using direct Enzyme‐Linked Immunosorbent Assay (ELISA). Briefly, the tissue lysates in PBS were coated on 96‐well microtiter plates and then blocked with 1% BSA for 1 h at 37°C. After washing, each primary detection antibody was added at a dilution of 10,000–20,000x in blocking buffer, followed by incubation at 37°C for 1 h. Subsequently, an HRP‐conjugated secondary antibody was added at a dilution of 15,000x in blocking buffer and incubated for 1 h at 37°C. After another washing step, TMB substrate solution was added, and the plates were incubated at 37°C for 20 min in the dark. The enzyme reaction was stopped by adding the stop solution, and absorbance was measured at 450 nm using a microplate reader for data analysis. All antibodies are described in the Table S2.

### 2.8 Measurement of Serum INHBA, MMP7, PSAT1, SLC7A5, TGFBI, CEA, and CA19‐9 Protein Concentrations

We fractionated the whole blood samples obtained from 200 CRC patients and 100 healthy subjects before any surgical procedures. The sera from both healthy individuals and CRC patients were immediately stored at −80°C after sampling to preserve protein integrity. Prior to the assay, the samples were retrieved from storage and thawed at 4°C. Protein concentrations of INHBA (Cat No. HUDL01534), MMP7 (Cat. No. HUFI00209), PSAT1 (Cat. No. HUDL02426), SLC7A5 (Cat. No. HUDL01685), TGFBI (Cat. No. HUDL02871), CEA (Cat. No. HUFI00080), and CA19‐9 (Cat. No. HUES01800) in serum were determined using specific ELISA kits from AssayGenie (Dublin, Ireland), following the manufacturer's instructions. To ensure unbiased results, serum samples were randomized, and investigators were blinded to the group assignment during analysis. The intra‐assay and inter‐assay coefficients of variation (CVs) for all biomarkers were below 10% and 15%, respectively, indicating high precision and reproducibility. Quality control samples with known concentrations of each biomarker were included in each run to monitor assay performance and validate the results. Additionally, each serum sample was analyzed in duplicate to ensure accuracy. Standard curves were created for each biomarker to precisely determine protein concentrations.

### 2.9 Defining INHBA, MMP7, PSAT1, SLC7A5, TGFBI, CEA, and CA19‐9 Cutoff Value in ROC Analysis

To further evaluate the diagnostic value of the selected markers based on dichotomous classification, we conducted receiver‐operator characteristic (ROC) curve using the method introduced by Hanley and McNeil [12]. The optimum cutoff values of INHBA, MMP7, PSAT1, SLC7A5, TGFBI, CEA, and CA19‐9 for the diagnosis of colorectal cancers were defined using the ROC curve and Youden's index in sera obtained from healthy individuals and CRC patients. Ranges of sensitivity and specificity values were calculated based on the cutoff value of the serum INHBA, MMP7, PSAT1, SLC7A5, TGFBI, CEA, and CA19‐9 protein concentration. The serum INHBA, MMP7, PSAT1, SLC7A5, TGFBI, CEA, and CA19‐9 measurements in CRC patients were used to determine the appropriate cutoff value of INHBA, MMP7, PSAT1, SLC7A5, TGFBI, CEA, and CA19‐9 for diagnosing CRC in the ethnic population studied. The sensitivity (true positive fraction, TPF), specificity (true negative fraction, TNF), false‐negative fraction (FNF), false‐positive fraction (FPF), positive predictive value (PPV), negative predictive value (NPV), accuracy, positive likelihood ratio (LR+), negative likelihood ratio (LR‐), and diagnostic odds ratio (DOR) for the cutoff value were calculated according to previously described method [13, 14, 15]. We defined the cutoff value of serum INHBA, MMP7, PSAT1, SLC7A5, TGFBI, CEA, and CA19‐9 concentration by maximizing the overall prediction performance with the Youden's J index {J(χ) = (sensitivity + specificity—1)} based on the ROC analysis.

### 2.10 Statistical Analysis

We examined serum or tissues INHBA, MMP7, PSAT1, SLC7A5, TGFBI, CEA, and CA19‐9 protein concentrations in duplicate to verify the reproducibility of the results. All statistical tests were performed using MedCalc (MedCalc Software, Mariakerke, Belgium), Graphpad Prism (GraphPad Software Inc., San Diego, CA), and SAS (SAS Institute; Cary, NC, USA). As serum INHBA, MMP7, PSAT1, SLC7A5, TGFBI, CEA, and CA19‐9 protein concentrations in healthy controls and CRC patients showed markedly left‐skewed distributions in both groups, so we presented the results as medians and interquartile ranges (IQR, 25th and 75th percentiles), and differences between groups were compared Mann–Whitney U‐test. Diagnostic performance was evaluated using the McNemar test. A *p* value less than 0.05 is considered as statistically significant.

## 3 Results

### 3.1 Identification of Extracellular Region and Space Genes as Potential Biomarkers for Colorectal Cancer

**FIGURE 1:**
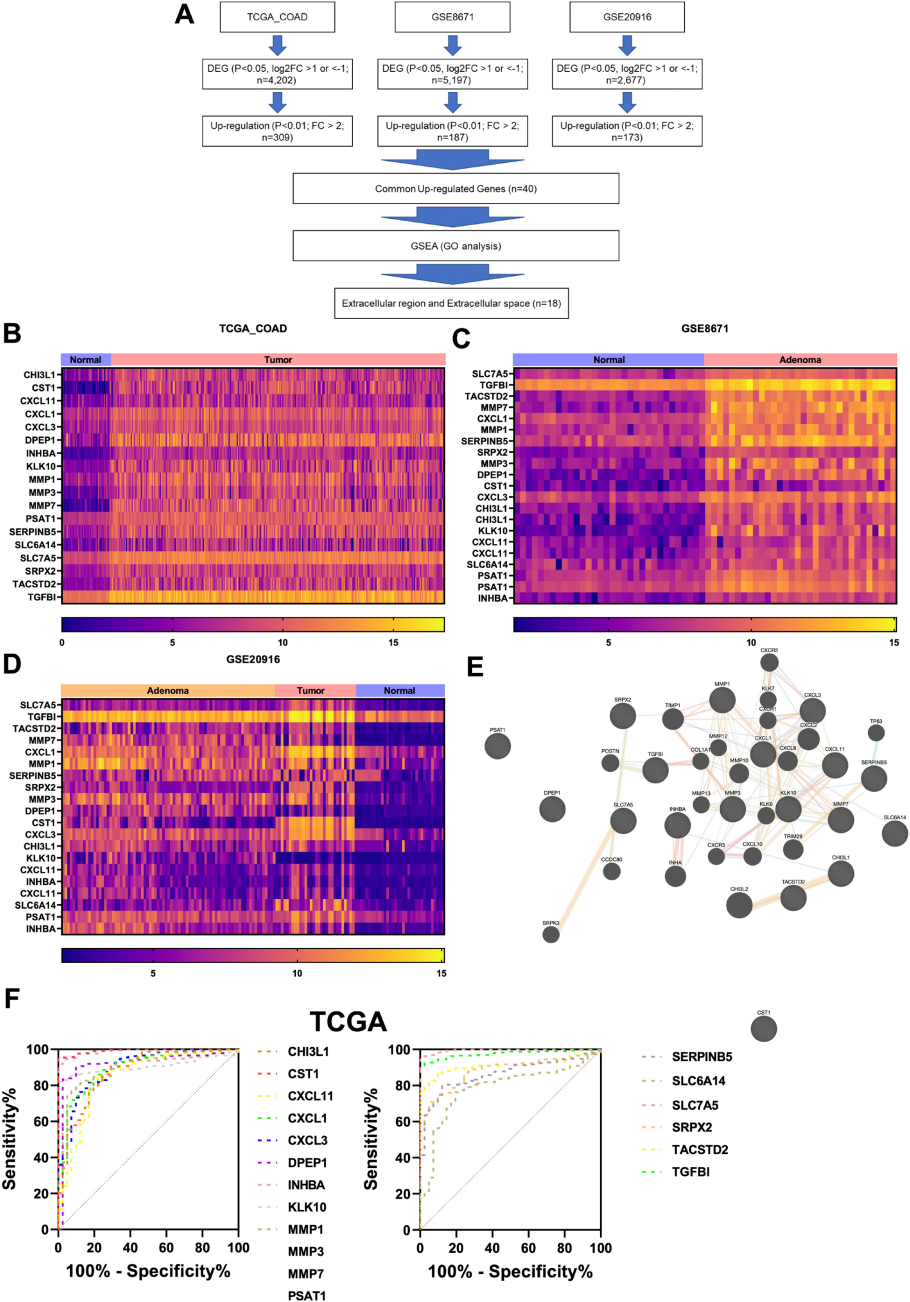
Identification of potential biomarkers for CRC in the extracellular region and space. (A) A flowchart illustrating the process of transcriptome analysis process encompassing data from TCGA, GSE87194, and GSE12916 datasets. The workflow includes data acquisition, normalization, identifying common up‐regulated genes, and GO analysis. (B–D) Heatmap visualization depicting distinct cluster differences in gene expression between CRC and normal samples for the 18 identified biomarkers across three datasets: TCGA_COAD (B), GSE8671 (C), and GSE20916 (D). (E) GeneMania network analysis reveals interactions between the 18 identified hubs, with central genes such as MMP3, KLK10, INHBA, TGFBI, CXCL1, MMP7, and MMP1 being involved in extensive cross‐talk, especially within the cardiac region. (F) A ROC curve for the TCGA_COAD dataset displays the performance of a model in distinguishing between different conditions by considering specificity and sensitivity.

We systematically examined gene expression profiles across three distinct datasets: TCGA_COAD, GSE8671, and GSE20916 to identify potential biomarkers for colorectal cancer. Utilizing stringent criteria of a *p* value threshold of ≤ 0.05 and log_2_ fold change greater than 1 or less than −1, we identified a total of 4202, 5197, and 2677 DEGs, respectively. Further refinement led us to identify 309, 187, and 173 active candidates, respectively, with a *p* value of 0.01 or less and a log_2_ fold change of 2 higher across the datasets. Notably, through comprehensive GO analysis, we identified 18 prominent entities predominantly increasing in abundance, residing within the extracellular region and space (Figure 1A). Further analysis revealed distinct cluster differences between CRC and normal samples for these 18 genes, as depicted through heatmap visualization across all three datasets (Figure 1B–D). This vividly portrayed the substantial upregulation of all 18 entities in CRC, underscoring their potential as discriminatory biomarkers. Further exploration utilizing the GeneMania program unveiled a network of interactions among the 18 identified hubs, with genes such as MMP3, KLK10, INHBA, TGFBI, CXCL1, MMP7, and MMP1 found to be centrally located and engaging in extensive cross‐talk with other nodes, particularly within the cardiac region, thereby elucidating potential functional relationships (Figure 1E). Subsequently, we conducted ROC curve analysis at the mRNA level for each entity to assess their ability to distinguish between normal and CRC samples. Remarkably, the majority of entities demonstrated robust discriminatory power, with Area Under the Curve (AUC) values exceeding 0.8 across the TCGA_COAD, GSE8671, and GSE20916 datasets (Figure 1F and Table 1), indicating their potential as effective diagnostic markers. In essence, our findings underscore the potential significance of these 18 identified biomarkers, residing within the extracellular milieu, as promising candidates for diagnosing CRC patients. The consistent upregulation and robust discriminatory power across multiple datasets not only signify their importance in advancing our understanding of CRC pathology but also highlight their potential to enhance the accuracy of diagnostic strategies, thereby potentially improving patient outcomes.

**TABLE 1:** AUC values for mRNA entities distinguishing normal and CRC samples across TCGA_COAD, GSE8671, and GSE20916 datasets.

Gene name	TCGA			GSE8671			GSE20671		
	AUC	95% CI	*p*	AUC	95% CI	*p*	AUC	95% CI	*p*
CHI3L1	0.8762	0.8162–0.9362	< 0.0001	0.9531	0.9083–0.9979	< 0.0001	0.8821	0.8029–0.9613	< 0.0001
CST1	0.9925	0.9862–0.9988	< 0.0001	0.8857	0.8021–0.9694	< 0.0001	0.9335	0.8958–0.9712	< 0.0001
CXCL11	0.8495	0.7794–0.9196	< 0.0001	0.9365	0.8693–1.000	< 0.0001	0.8916	0.8317–0.9516	< 0.0001
CXCL1	0.8955	0.8451–0.9459	< 0.0001	0.9453	0.8850–1.000	< 0.0001	0.8794	0.8240–0.9349	< 0.0001
CXCL3	0.8947	0.8427–0.9467	< 0.0001	0.9551	0.9085–1.000	< 0.0001	0.8898	0.8342–0.9454	< 0.0001
DPEP1	0.9191	0.8685–0.9697	< 0.0001	0.9854	0.9648–1.000	< 0.0001	0.8990	0.8485–0.9496	< 0.0001
INHBA	0.9903	0.9826–0.9981	< 0.0001	0.9775	0.9454–1.000	< 0.0001	0.9290	0.8841–0.9739	< 0.0001
KLK10	0.9056	0.8740–0.9372	< 0.0001	0.9814	0.9497–1.000	< 0.0001	0.8034	0.7343–0.8724	< 0.0001
MMP1	0.8933	0.8507–0.9358	< 0.0001	0.9893	0.9710–1.000	< 0.0001	0.9057	0.8498–0.9616	< 0.0001
MMP3	0.9036	0.8626–0.9447	< 0.0001	0.9736	0.9400–1.000	< 0.0001	0.9674	0.9415–0.9933	< 0.0001
MMP7	0.9814	0.9686–0.9942	< 0.0001	1.000	1.000–1.000	< 0.0001	0.9277	0.8834–0.9719	< 0.0001
PSAT1	0.9699	0.9524–0.9874	< 0.0001	0.9980	0.9928–1.000	< 0.0001	0.9287	0.8873–0.9702	< 0.0001
SERPINB5	0.8632	0.8186–0.9077	< 0.0001	0.9932	0.9812–1.000	< 0.0001	0.7530	0.6568–0.8493	< 0.0001
SLC6A14	0.7864	0.7208–0.8521	< 0.0001	0.8223	0.7223–0.9222	< 0.0001	0.8254	0.7510–0.8998	< 0.0001
SLC7A5	0.9951	0.9902–0.9999	< 0.0001	0.9951	0.9856–1.000	< 0.0001	0.9849	0.9696–1.000	< 0.0001
SRPX2	0.8814	0.8406–0.9221	< 0.0001	0.9951	0.9842–1.000	< 0.0001	0.9372	0.8975–0.9769	< 0.0001
TACSTD2	0.9120	0.8810–0.9431	< 0.0001	1.000	1.000–1.000	< 0.0001	0.9539	0.9200–0.9878	< 0.0001
TGFBI	0.9744	0.9592–0.9896	< 0.0001	1.000	1.000–1.000	< 0.0001	0.9287	0.8844–0.9731	< 0.0001

### 3.2 Validation of Selected Genes and Proteins as Biomarkers for Colorectal Cancer Tissues

**FIGURE 2:**
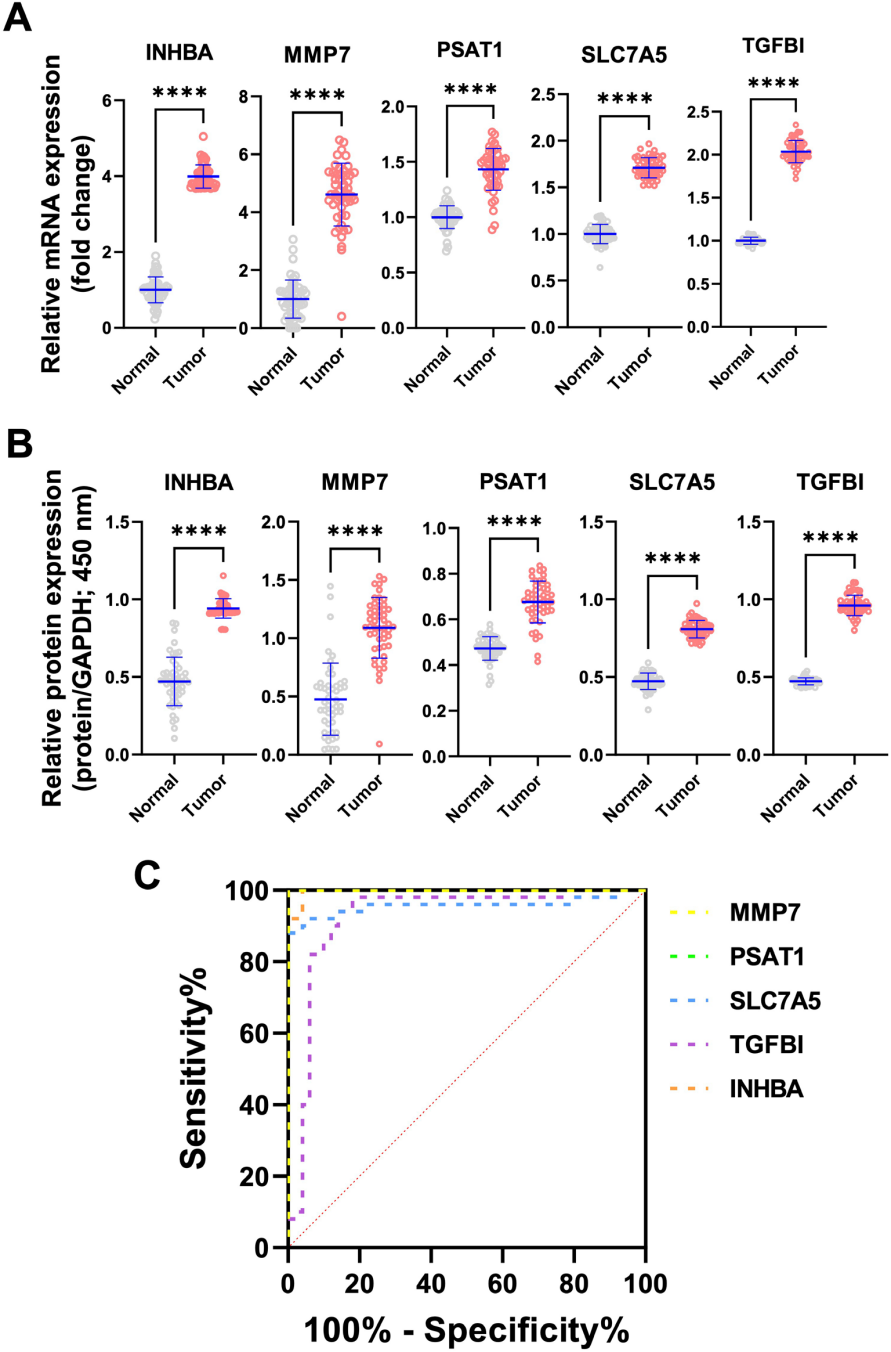
Comparative analysis of gene expression and protein levels in CRC tissues. (A) The relative mRNA expression levels of INHBA, MMP7, PSAT1, SLC7A5, and TGFBI in 60 CRC tissues compared to normal tissues. Each dot represents an individual sample. (B) The relative protein expression levels of INHBA, MMP7, PSAT1, SLC7A5, and TGFBI in CRC tissues compared to normal tissues. Each dot represents an individual sample. (C) A ROC curve showing the sensitivity and specificity of each marker (INHBA, MMP7, PSAT1, SLC7A5, TGFBI) for distinguishing between CRC tissues and normal tissues.

Next, among the 18 genes discovered in the three dataset analyses, we prioritized genes with high AUC values in ROC curve analysis. Consequently, we selected five genes—INHBA, MMP7, PSAT1, SLC7A5, and TGFBI—to evaluate their mRNA expression levels in normal colon tissues compared to their corresponding cancer tissues. Upon confirmation of mRNA expression via real‐time quantitative polymerase chain reaction (qPCR), all five genes exhibited significantly increased expression in 60 CRC tissues compared to normal tissues (Figure 2A). Furthermore, we evaluated the protein expression of these genes using direct ELISA, observing a similar pattern of significantly elevated protein levels in CRC tissue compared to normal tissue, mirroring the mRNA expression findings (Figure 2B). Subsequently, we conducted ROC curve analysis based on the protein expression levels of these selected biomarkers. Strikingly, the AUC values were 0.9968 for INHBA, 0.9224 for MMP7, 0.9564 for PSAT1, 1.000 for SLC7A5, and 1.000 for TGFBI, respectively (Figure 2C and Table 2). These findings underscore the robust diagnostic potential of these proteins as effective biomarkers for CRC, emphasizing their utility in enhancing diagnostic accuracy and potentially improving patient outcomes.

**TABLE 2:** AUC values of protein expression in CRC tissues for selected biomarkers from ROC curve analysis.

Gene name	AUC	95% CI	*p*
INHBA	0.9968	0.9911–1.000	< 0.0001
MMP7	0.9224	0.8586–0.9862	< 0.0001
PSAT1	0.9564	0.9082–1.000	< 0.0001
SLC7A5	1.000	1.000–1.000	< 0.0001
TGFBI	1.000	1.000–1.000	< 0.0001

### 3.3 Analysis of Protein Concentration in Serum Samples From Normal Individuals and Colorectal Cancer Patients

**FIGURE 3:**
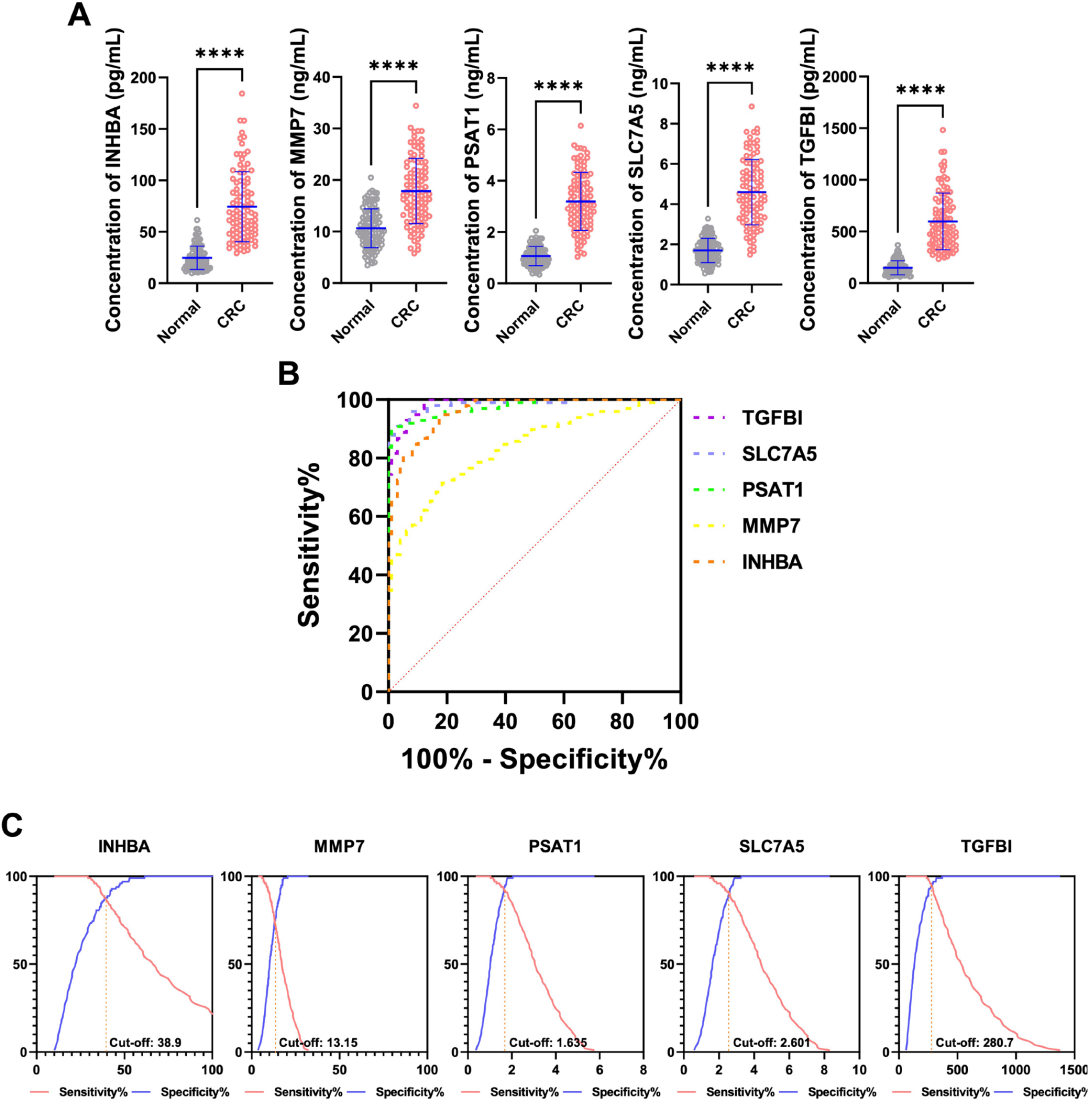
Serum concentrations of candidate biomarkers in CRC patients and healthy controls. (A) The concentration of INHBA, MMP7, PSAT1, SLC7A5, and TGFBI in serum samples from 200 CRC patients and 100 healthy individuals. (B) A ROC curve showing the sensitivity and specificity of each gene (TGFBI, SLC7A5, PSAT1, MMP7, INHBA) in distinguishing between CRC patients and healthy individuals. (C) ROC curves with corresponding cutoff values for INHBA, MMP7, PSAT1, SLC7A5, and TGFBI.

To evaluate the potential value of serum INHBA, MMP7, PSAT1, SLC7A5, and TGFBI protein concentrations as innovative biomarkers for early detection of CRC, we conducted a study with 200 CRC patients and 100 healthy controls. Our findings, as shown in Figure 3A and Figure S1A, demonstrate significantly elevated serum levels of INHBA (median: 66.15 pg/mL, IQR: 47.21–97.84 pg/mL), MMP7 (median: 17.10 ng/mL, IQR: 47.21–97.84 ng/mL), PSAT1 (median: 3.053 ng/mL, IQR: 2.314–4.008 ng/mL), SCL7A5 (median: 4.396 ng/mL, IQR: 3.331–5.771 ng/mL), and TGFBI (median: 531.8 pg/mL, IQR: 379.6–786.6 pg/mL) proteins in CRC patients compared to healthy individuals (median: 22.05 pg/mL, IQR: 15.74–32.61 pg/mL for INHBA; median: 10.18 ng/mL, IQR: 7.712–13.36 ng/mL for MMP7; median: 1.018 ng/mL, IQR: 0.771–1.336 ng/mL for PSAT1; median: 1.628 pg/mL, IQR: 1.234–2.137 pg/mL for SCL7A5; median: 132.3 pg/mL, IQR: 94.42–195.7 pg/mL for TGFBI). This contrasts with conventional markers CEA (median: 3.307 ng/mL, IQR: 2.296–4.892 ng/mL in CRC and median: 1.654 ng/mL, IQR: 1.086–2.446 ng/mL in healthy controls) and CA19‐9 (median: 11.23 IU/mL, IQR: 8.514–14.75 IU/mL in CRC and median: 8.141 IU/mL, IQR: 6.169–10.36 IU/mL in healthy controls), which also showed elevation but to a lesser extent (Figure 3A and Figure S1A).

Furthermore, ROC curve analysis (Figure 3B, Table 3, Figure S1B, and Table S3) revealed robust discriminatory ability, with impressive AUC values of 0.9610 for INHBA, 0.8361 for MMP7, 0.9767 for PSAT1, 0.9846 for SLC7A5, and 0.9869 for TGFBI. These values indicate their superior diagnostic potential compared to CEA (AUC: 0.8561) and CA19‐9 (AUC: 0.7315).

**TABLE 3:** AUC values for serum biomarkers INHBA, MMP7, PSAT1, SLC7A5, and TGFBI.

Gene Name	AUC	95% CI	*p*
INHBA	0.9610	0.9389–0.9830	< 0.0001
MMP7	0.8361	0.7808–0.8914	< 0.0001
PSAT1	0.9767	0.9588–0.9945	< 0.0001
SLC7A5	0.9846	0.9700–0.9992	< 0.0001
TGFBI	0.9869	0.9764–0.9973	< 0.0001

Determining optimal cutoff values for CRC diagnosis (Figure 3C, Table 4, Figure S1C, and Table S4) revealed values of 38.9 pg/mL for INHBA, 13.15 ng/mL for MMP7, 1.635 ng/mL for PSAT1, 2.601 ng/mL for SLC7A5, 280.7 pg/mL for TGFBI, 2.357 ng/mL for CEA, and 9.571 IU/mL for CA19‐9. Comparing these biomarkers, INHBA, MMP7, PSAT1, SLC7A5, and TGFBI demonstrated superior diagnostic accuracy and odds ratios over the traditional markers CEA and CA19‐9. For instance, INHBA showed a sensitivity of 84.69% and a specificity of 91.83%, significantly higher than CEA's sensitivity of 72.27% and specificity of 71.43%. Additionally, the Youden's index for INHBA is 76.53% compared to 43.71% for CEA and 31.64% for CA19‐9, emphasizing the stronger diagnostic potential of these novel biomarkers. This substantial improvement underscores the high diagnostic accuracy and diagnostic odds ratios (DOR) of INHBA, MMP7, PSAT1, SLC7A5, and TGFBI in CRC detection. We advocate for their integration into non‐invasive diagnostic approaches, marking a notable advancement in CRC diagnosis.

**TABLE 4:** Optimal cutoff values and diagnostic performance metrics of biomarkers for CRC diagnosis.

	INHBA	MMP7	PSAT1	SLC7A5	TGFBI
Sensitivity	0.846939	0.744898	0.928571	0.918367	0.887755
Specificity	0.918367	0.744898	0.897959	0.928571	0.938776
PPV	0.846939	0.744898	0.928571	0.918367	0.887755
NPV	0.918367	0.744898	0.897959	0.928571	0.938776
LR+	10.375	2.92	9.1	12.85714	14.5
LR−	0.166667	0.342466	0.079545	0.087912	0.119565
DOR	1.729167	1	0.723864	1.130298	1.733696
Accuracy	0.5	0.5	0.5	0.5	0.5
Youden's index	0.765306	0.489796	0.826531	0.846939	0.826531

## 4 Discussion

The findings presented in this study provide valuable insights into the identification and validation of potential biomarkers for CRC across different molecular levels, emphasizing their diagnostic and prognostic significance. The urgency of developing effective biomarkers for CRC diagnosis is underscored by the disease's significant global burden and the imperative for early detection to improve patient outcomes.

The systematic examination of gene expression profiles across multiple datasets allowed us to identify 18 promising biomarkers predominantly localized within the extracellular milieu. These genes exhibited consistent upregulation in CRC samples compared to normal tissues, indicating their potential as differentiating biomarkers for CRC diagnosis. Moreover, the subsequent analysis of protein expression levels in CRC tissues further validated the diagnostic utility of selected biomarkers, namely INHBA, MMP7, PSAT1, SLC7A5, and TGFBI. The high expression levels of these proteins in CRC tissues highlight their potential as effective diagnostic markers for identifying CRC. Furthermore, our investigation includes the analysis of protein concentrations in serum samples between CRC patients and the control group. The significantly elevated serum levels of INHBA, MMP7, PSAT1, SLC7A5, and TGFBI proteins in CRC patients compared to healthy individuals suggest their potential as non‐invasive biomarkers for CRC detection. The robust discriminatory ability of these proteins, as evidenced by high AUC values in ROC curve analysis, highlights their promising diagnostic efficacy in CRC. INHBA, a member of the TGF‐beta superfamily, has been implicated in promoting tumor growth, invasion, and metastasis in various cancers, including CRC [16, 17, 18, 19]. Its upregulation in CRC tissues suggests its involvement in driving tumorigenesis and disease progression [20, 21, 22]. Similarly, MMP7, a matrix metalloproteinase, is known to contribute to tumor invasion and metastasis by degrading extracellular matrix components [23, 24, 25]. Its elevated expression in CRC tissues underscores its role in facilitating cancer cell invasion and metastatic dissemination [26, 27, 28, 29]. PSAT1, an enzyme involved in serine biosynthesis, has been associated with promoting cell proliferation and survival in cancer cells [30, 31, 32]. Its increased expression in CRC tissues may support tumor cell growth and survival, highlighting its potential as a biomarker for CRC progression [33, 34]. SLC7A5, a member of the solute carrier family, plays a critical role in amino acid transport, which is essential for cancer cell metabolism and growth [35, 36, 37]. Its upregulation in CRC tissues suggests its involvement in supporting the metabolic demands of proliferating cancer cells [38, 39]. TGFBI, an extracellular matrix protein, has been implicated in modulating cell adhesion, migration, and proliferation in cancer [40, 41, 42]. Its elevated expression in CRC tissues may contribute to tumor progression and metastasis by promoting cancer cell motility and invasion [43, 44].

However, despite the promising results, several limitations warrant further consideration. Firstly, this study primarily focused on identifying and validating potential biomarkers based on gene expression and protein levels. Further functional characterization of these biomarkers can clarify their roles in CRC pathogenesis and progression. Additionally, although the selected biomarkers demonstrate high diagnostic accuracy, validation in larger patient cohorts is essential to establish their clinical utility and generalizability. Furthermore, expanding the sample size to encompass a more diverse population would enhance the robustness and applicability of the findings across different demographic and racial groups. Moreover, although the initial findings are encouraging, it is imperative to discuss plans or the necessity for long‐term clinical studies to comprehensively validate these biomarkers before they can be widely adopted in clinical practice.

In conclusion, the identification and validation of biomarkers such as INHBA, MMP7, PSAT1, SLC7A5, and TGFBI represent a significant advancement in CRC diagnosis and patient care. These novel biomarkers offer promising prospects for enhancing diagnostic accuracy and tailoring personalized treatment strategies, potentially improving patient outcomes. Compared with established biomarkers such as CEA and CA19‐9, these newly identified biomarkers demonstrate superior performance in terms of specificity, sensitivity, and prognostic value. The integration of these tools into clinical practice could revolutionize CRC management by providing clinicians with more precise and reliable diagnostic options. However, further research efforts are needed to clarify the functional roles of these biomarkers, validate their clinical usefulness in larger cohorts, and explore new diagnostic methods. Ultimately, the goal is to optimize patient care and outcomes in CRC.

## Author Contributions

**Minha Lea Yoon:** data curation (equal), investigation (equal), writing – review and editing (equal). **Hyelim Chun:** formal analysis (equal), investigation (equal), visualization (equal). **HyunJu Lee:** formal analysis (equal), investigation (equal). **WooJeong Seo:** formal analysis (equal), investigation (equal). **Jung Young Lee:** supervision (equal), writing – review and editing (equal). **Jung Hwan Yoon:** conceptualization (lead), data curation (equal), formal analysis (equal), supervision (lead), visualization (lead), writing – original draft (lead).

## Ethics Statement

All experiments were conducted in accordance with the Declaration of Helsinki, and the study was approved by the Institutional Review Board of the Catholic University of Korea College of Medicine (MC15SISI0015) and Kangnam St Peter's Hospital (SPH20‐24‐001). Anonymized serum, CRC tissues, and clinical data were provided through the Korea Biobank network of Chonnam National University Hwasun Hospital and Catholic University Gangnam St Mary's Hospital. The requirement for informed consent has been waived.

## Conflicts of Interest

The authors declare no conflicts of interest.

## Supporting information


**Figure S1.** Serum concentrations of established biomarkers in CRC patients and healthy controls.

**Table S1.** Primer sequence used in this study.
**Table S2.** Antibody used in this study.
**Table S3.** AUC values for established biomarkers CEA and CA19‐9.

## Data Availability

The data, materials, and reagents supporting the findings of this study will be made available upon reasonable request.
