# Supplementary material for: Identification and Validation of Serum Biomarkers to Improve Colorectal Cancer Diagnosis
Source: Cancer Med. 2024 Dec 4;13(23):e70460. doi: 10.1002/cam4.70460 (PMC11615507; doi:10.1002/cam4.70460)
Supplement: Supplementary file 1 — Figure S1. Serum concentrations of established biomarkers in CRC patients and healthy controls. [file CAM4-13-e70460-s001.docx]

**
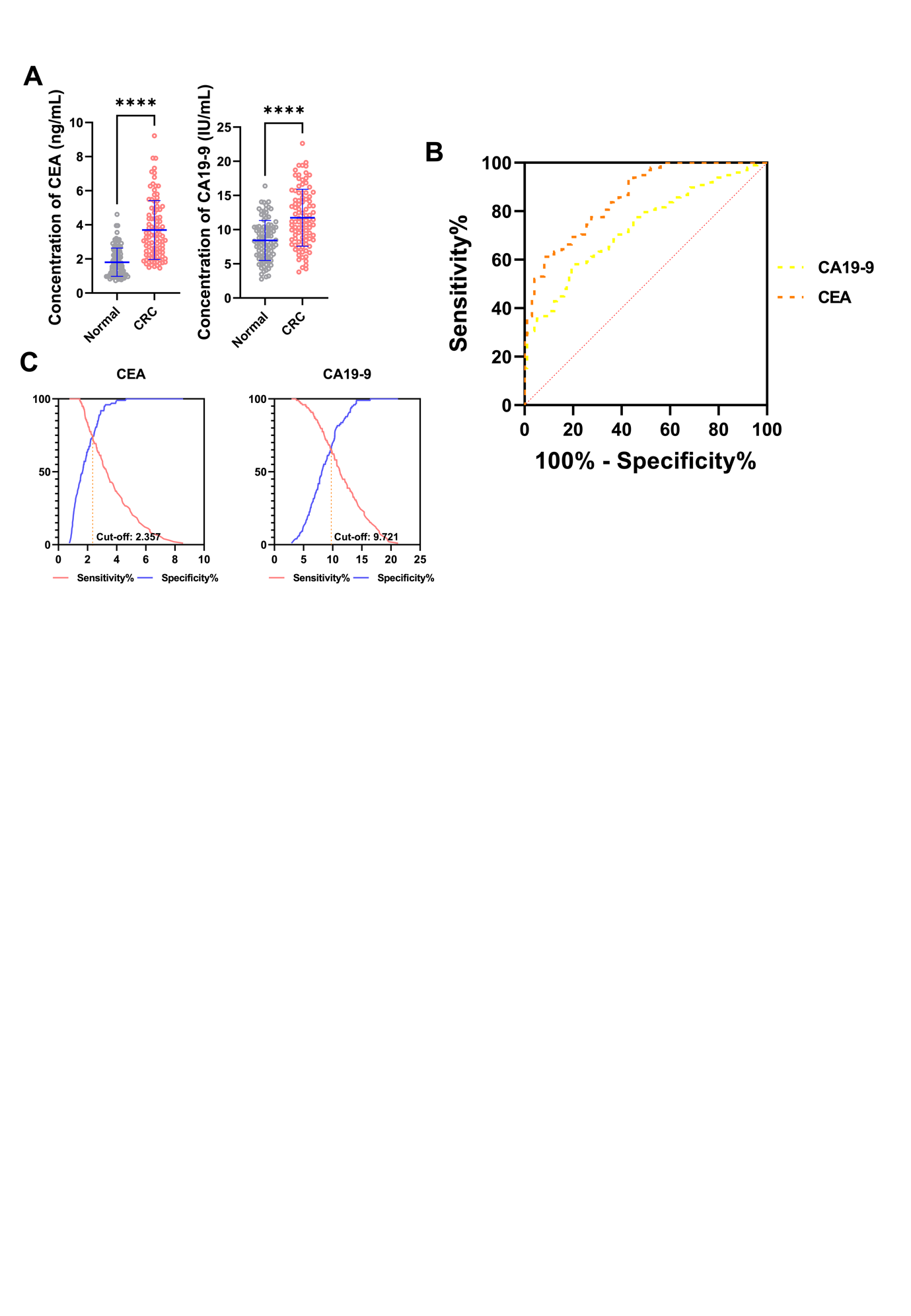
**

**Figure S1. Serum concentrations of established biomarkers in CRC patients and healthy controls.** A) The concentration of CEA and CA19-9 in serum samples from 200 CRC patients and 100 healthy individuals. B) A ROC curve showing the sensitivity and specificity of CEA and CA19-9 in distinguishing between CRC patients and healthy individuals. C) ROC curves with corresponding cut off values for CEA and CA19-9.
