# Supplementary material for: Identification and Validation of Serum Biomarkers to Improve Colorectal Cancer Diagnosis
Source: Cancer Med. 2024 Dec 4;13(23):e70460. doi: 10.1002/cam4.70460 (PMC11615507; doi:10.1002/cam4.70460)
Supplement: Supplementary file 2 — Table S1. Primer sequence used in this study. Table S2. Antibody used in this study. Table S3. AUC values for established biomarkers CEA and CA19‐9. [file CAM4-13-e70460-s002.docx]

Table S1. Primer sequence used in this study.

| Gene name | Sequence |
| --- | --- |
| INHBA | F: 5’-GGTATGTGGAGATAGAGGATGAC-3’ |
|  | R: 5’-TCCTGGCTGTTCCTGACTC-3’ |
| MMP7 | F: 5’-CATGAGTGAGCTACAGTGGGA-3’ |
|  | R: 5’-CTATGACGCGGGAGTTTAACAT-3’ |
| PSAT1 | F: 5’-GTCCAGTGGAGCCCCAAAA-3’ |
|  | R: 5’-TGCCTCCCACAGACCTATGC-3’ |
| SLC7A5 | F: 5’-CCGTGAACTGCTACAGCGT-3’ |
|  | R: 5’-CTTCCCGATCTGGACGAAGC-3’ |
| TGFBI | F: 5’-CAGCTACGAGTGCTGTCCTG-3’ |
|  | R: 5’-TACAGCTGAGTGGTGGTGGA-3’ |
| ACTB | F: 5’-AGTTGCGTTACACCCTTTCTTGAC-3’ |
|  | R: 5’-GCTCGCTCCAACCGACTGC-3’ |

Table S2. Antibody used in this study.

| Protein name | Company | Cat No. |
| --- | --- | --- |
| INHBA | Abcam | Ab56057 |
| MMP7 | Cell signaling Tech. | 71031 |
| PSAT1 | Proteintech Group Inc. | 10501-1-AP |
| SLC7A5 | Cell signaling Tech. | 5347 |
| TGFBI | Proteintech Group Inc. | 10188-1-AP |
| GAPDH | Proteintech Group Inc. | 80570-1-RR |
| Anti-rabbit IgG, HRP-linked antibody | Cell signaling Tech. | 7074 |

Table S3. AUC values for established biomarkers CEA and CA19-9.

| **Protein Name** | **AUC** | **95% CI** | **P value** |
| --- | --- | --- | --- |
| CEA | 0.8561 | 0.8063-0.9059 | <0.0001 |
| CA19-9 | 0.7315 | 0.6619-0.8010 | <0.0001 |

Table S4. Optimal cutoff values and diagnostic performance metrics for established biomarkers CEA and CA19-9.

|  | **CEA** | **CA19-9** |
| --- | --- | --- |
| Sensitivity | 0.722772 | 0.663366 |
| Specificity | 0.714286 | 0.653061 |
| PPV | 0.722772 | 0.663366 |
| NPV | 0.736842 | 0.673684 |
| LR+ | 1.011881 | 1.01578 |
| LR- | 0.357143 | 0.484375 |
| DOR | 2.833267 | 2.097094 |
| Accuracy | 0.729592 | 0.668367 |
| Youden's index | 0.437058 | 0.316428 |
